# *In vivo* regeneration of interspecies chimeric kidneys using a nephron progenitor cell replacement system

**DOI:** 10.1038/s41598-019-43482-2

**Published:** 2019-05-06

**Authors:** Toshinari Fujimoto, Shuichiro Yamanaka, Susumu Tajiri, Tsuyoshi Takamura, Yatsumu Saito, Kei Matsumoto, Kentaro Takase, Shohei Fukunaga, Hirotaka James Okano, Takashi Yokoo

**Affiliations:** 1 Division of Nephrology and Hypertension, Department of Internal Medicine, The Jikei University School of Medicine, 3-25-8, Nishi-Shimbashi, Minato-ku, Tokyo, 105-8461 Japan; 2 Division of Regenerative Medicine, The Jikei University School of Medicine, 3-25-8, Nishi-Shimbashi, Minato-ku, Tokyo, 105-8461 Japan; 3 Division of Cardiology and Nephrology, Department of Internal Medicine, Shimane University Faculty of Medicine, Izumo, Shimane 693-8501 Japan

**Keywords:** Nephrology, Developmental biology

## Abstract

Kidney regeneration is expected to be a new alternative treatment to the currently limited treatments for chronic kidney disease. By transplanting exogeneous nephron progenitor cells (NPCs) into the metanephric mesenchyme of a xenogeneic foetus, we aimed to regenerate neo-kidneys that originate from transplanted NPCs. Previously, we generated a transgenic mouse model enabling drug-induced ablation of NPCs (the Six2-iDTR mouse). We demonstrated that eliminating existing native host NPCs allowed their 100% replacement with donor mouse or rat NPCs, which could generate neo-nephrons on a culture dish. To apply this method to humans in the future, we examined the possibility of the *in vivo* regeneration of nephrons between different species via NPC replacement. We injected NPCs-containing rat renal progenitor cells and diphtheria toxin below the renal capsule of E13.5 metanephroi (MNs) of Six2-iDTR mice; the injected MNs were then transplanted into recipient rats treated with immunosuppressants. Consequently, we successfully regenerated rat/mouse chimeric kidneys in recipient rats receiving the optimal immunosuppressive therapy. We revealed a functional connection between the neo-glomeruli and host vessels and proper neo-glomeruli filtration. In conclusion, we successfully regenerated interspecies kidneys *in vivo* that acquired a vascular system. This novel strategy may represent an effective method for human kidney regeneration.

## Introduction

The increasing number of patients with chronic kidney disease is a serious public health issue. Likewise, end-stage renal disease is a leading cause of morbidity and mortality worldwide^1^. The global use of renal replacement therapy is expected to increase sharply in the next decade^2^. Considering quality of life and prognosis, kidney transplantation will be the first choice of treatment for renal disease^3^. However, the shortage of transplant organs is a serious global problem^4^. To overcome this problem, regenerative medicine will continue to emerge as a new treatment strategy.

Recently, research in kidney regeneration has made remarkable progress. By studying the developmental process of kidneys, the differential induction of nephron progenitor cells (NPCs)^5–7^ and ureteric buds (UBs)^8,9^ from human-induced pluripotent stem cells (iPSCs) has been achieved *in vitro*. Additionally, previous studies have reported the assembly of kidney organoids that recapitulate embryonic branching morphogenesis by reaggregating NPCs and UBs derived from pluripotent stem cells (PSCs)^8^. However, kidney organoids have no urine drainage system and are too small to function *in vivo*. Therefore, generating functional kidneys via this approach remains challenging.

As an alternative, previous studies have considered several methods of regenerating solid organs that are capable of functioning *in vivo* from transplanted exogenous cells by borrowing a xenogeneic development programme. One such method is blastocyst complementation. PSCs are injected into blastocysts that have undergone genetic manipulation in order to not generate a target organ; the PSCs then form the missing organ. Kidneys can be generated in an allogenic environment^10^; in addition, a pancreas can be generated in a xenogeneic environment^11,12^, as shown by blastocyst complementation in rodent models. However, because this method is based on systemic chimera formation, it is a serious ethical concern to develop chimera formation in host gametes or neural tissues other than the target organs. Conversely, we have reported on the organogenic niche method in which exogenous human mesenchymal stem cells were injected into the metanephric mesenchyme of xenogeneic rat foetuses and those human cells differentiated into kidneys^13–15^. This method is novel because stem cells, which have limited potency, are used as a cell source instead of PSCs. We used embryos from mid-to-late gestational stages instead of early embryos as a host because the transplantation of donor cells into developmental-stage-matched host tissue may be critical for the efficient engraftment of cells into chimeras^16^. The above discussion highlights the advantage of this method: chimera formation occurs only in the kidney, thereby avoiding ethical concerns.

In addition, we have recently developed a new organogenic niche method that is combined with eliminating host NPCs to increase the engraftment efficiency of donor cells^17^. In this new method, we used a Six2-iDTR transgenic mouse in which diphtheria toxin receptor (DTR) was specifically expressed on Six2-positive NPCs. We demonstrated that by administering diphtheria toxin (DT), the elimination of existing native host mouse NPCs allowed their 100% replacement with donor mouse or rat NPCs, which could generate neo-nephrons on a culture dish.

In the future, we will aim to regenerate human kidneys using pig foetuses as a bioreactor via our *in vivo* method to create larger regenerated kidneys. Hence, it is necessary to investigate whether this method can be applied between different species *in vivo*. However, such analyses have not yet been performed. In regenerated kidneys, the collecting duct system and interstitium both originate from the host animal, whereas the nephrons originate from the exogenous NPCs. We aimed to regenerate and grow interspecies chimeric kidneys *in vivo* using optimal immunosuppressants. In addition, we investigated whether regenerated kidneys could acquire a vascular system and develop filtration capacity. In the present study, we verified the possibility of *in vivo* regeneration of functional interspecies kidneys using an NPC replacement system in a mouse-rat rodent model.

## Results

### Regeneration of interspecies chimeric kidneys in immunodeficient hosts

**Figure 1:**
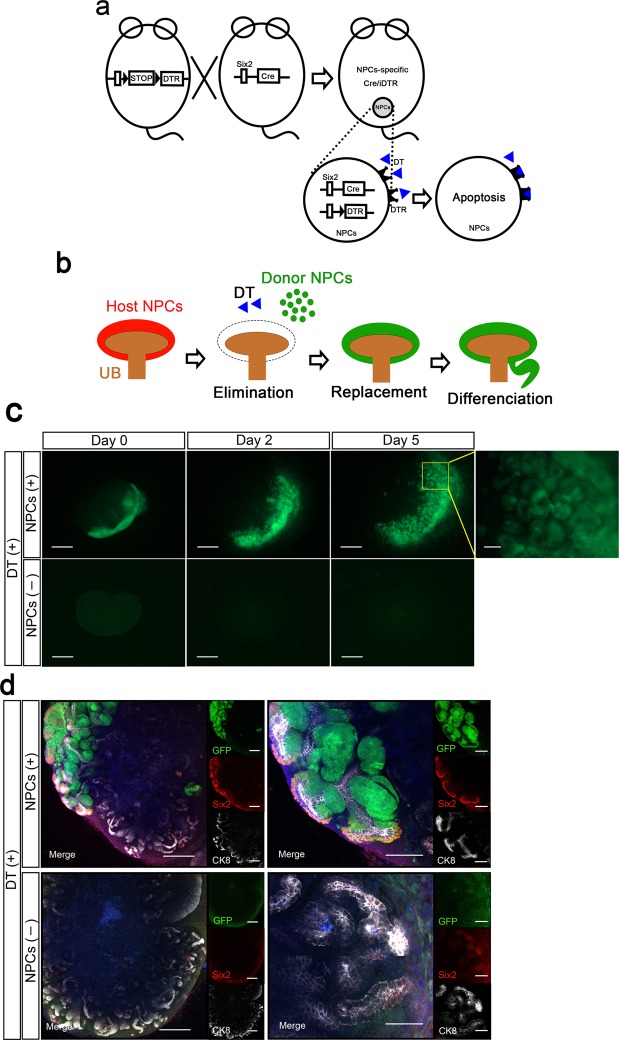
Six2-iDTR model for ablation and replacement of Six2-positive nephron progenitor cells. (**a**) Schematic of the Six2-Cre inducible iDTR system. DTR is specifically expressed on Six2-positive NPCs, and the administration of DT eliminates the NPCs. (**b**) Schematic of the drug-induced cell elimination system to exchange native NPCs with exogenous NPCs. (**c**) Organ culture of MNs isolated from E13.5 Six2-iDTR mouse embryos. Transplantation of rat NPCs-containing GFP-RPCs and daily administration of DT (upper column). No transplantation of rat GFP-RPCs and daily administration of DT (lower column). Scale bars, upper right column; 100 µm, others; 500 µm. (**d**) Immunostaining of MNs in (**c**). Transplanted rat GFP-NPCs were integrated into the cap mesenchyme with Six2 expression and epithelialised vesicles (upper column). When there was no NPC transplantation, Six2-GFP cells were eliminated, and poor branching of the UB was observed (lower column). Scale bars, upper and lower left column; 250 µm, upper right; 100 µm, lower right; 50 µm. Representative images of four MNs with RPC transplantation and two MNs without RPC transplantation from two independent experiments are indicated.

**Figure 2:**
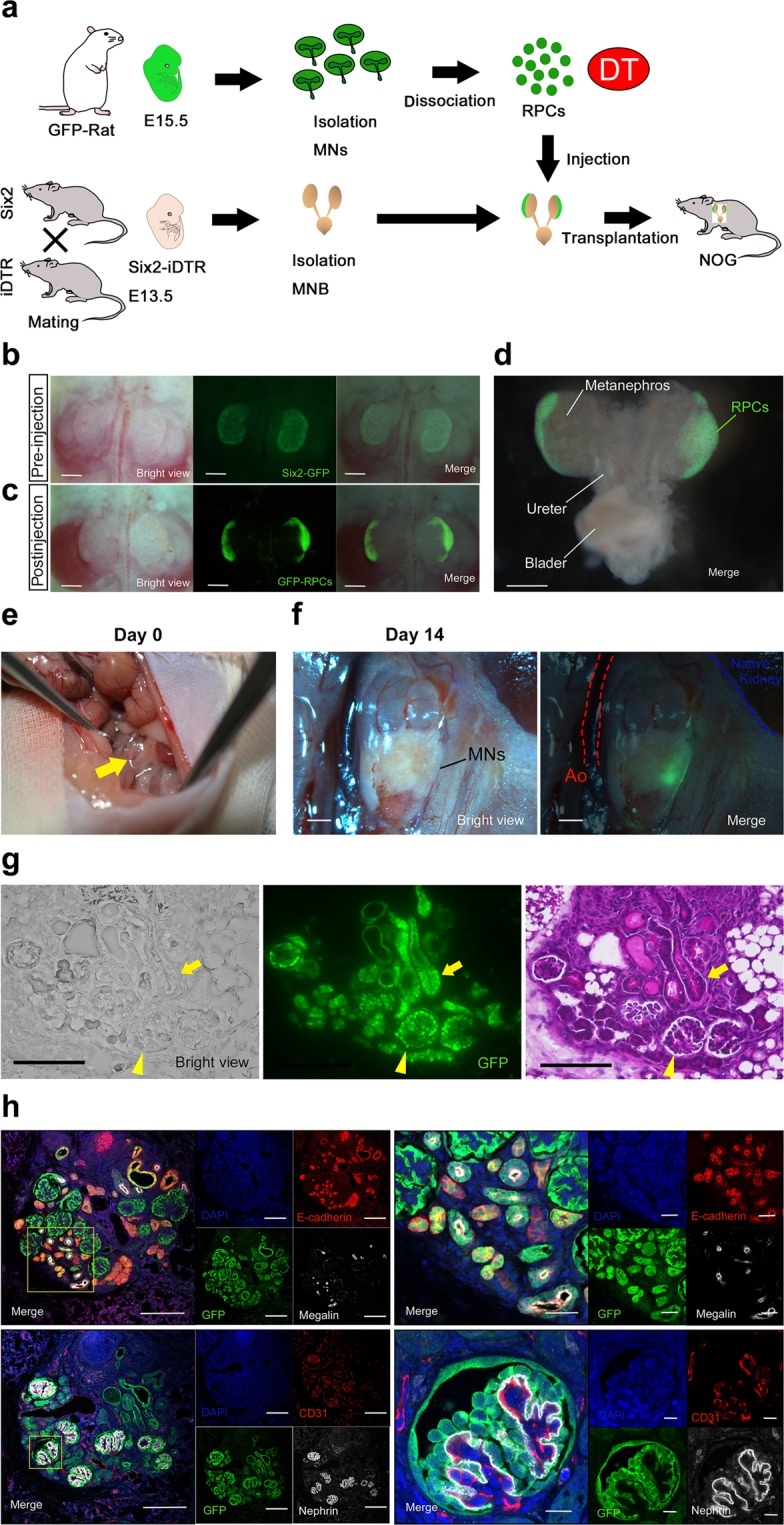
Regeneration of nephrons derived from transplanted rat NPCs in an adult NOG mouse using the nephron progenitor cell replacement system. (**a**) Schematic of the experimental protocol. (**b**) We observed Six2-GFP fluorescence in E13.5 MNs of the Six2-iDTR mice (scale bars, 500 µm). (**c**) DT and rat GFP-RPCs were injected under the renal capsule. The RPCs showed strong green fluorescent protein (GFP) expression (CAG promoter); therefore, we were unable to observe Six2-GFP (native promoter) under a fluorescence stereoscopic microscope alongside GFP-RPCs after transplantation (scale bars, 500 µm). (**d**) MNB was isolated from the Six2-iDTR mouse embryo (scale bar, 500 µm). (**e**) MNB-containing rat RPCs and DT were transplanted into the vicinity of the aorta of an adult NOG mouse. The arrow indicates MNB. (**f**) Fourteen days after transplantation, GFP expression was evaluated and identified (scale bars, 1 mm). (**g**) Frozen sections were created from the collected MNB. We observed GFP-positive glomerular (arrow head) and tubular structures (arrow) (left column; scale bars, 100 µm). HE staining demonstrated glomeruli (arrow head) and tubules (arrow) regenerated from the transplanted rat NPCs (right column; scale bar, 100 µm). (**h**) Frozen sections were analysed by immunostaining. We identified neo-glomeruli (lower column) and tubules (upper column) that originated from transplanted rat GFP-NPCs. Glomeruli had nephrin-positive epithelial cells lining CD31-positive vascular endothelial cells (right lower). All the cells originating in the NPCs were GFP-positive. Nephrin is a podocyte marker, megalin is a proximal tubular marker, E-cadherin is a distal tubular marker, and CD31 is an endothelial marker (scale bars, left upper: 100 µm, left lower; 100 µm, right upper: 25 µm, right lower: 10 µm). Representative images of five specimens from three independent transplantation experiments are indicated.

We attempted to regenerate rat nephrons using Six2-iDTR mouse metanephroi (MNs) as a biological scaffold (Fig. 1a,b). We isolated E15 MNs of GFP-rats and dissociated them to obtain NPCs-containing rat renal progenitor cells (RPCs). The obtained rat GFP-RPCs were injected below the renal capsule of E13.5 MNs of Six2-iDTR mice, followed by the culturing of injected MNs in a DT-supplemented medium. As we reported previously^17^, we eliminated existing native host mouse Six2-positive NPCs, allowing their complete replacement with donor rat NPCs *in vitro* (Fig. 1c,d; Supplementary Fig. S1). Regenerated chimeric kidneys will be rejected if they are placed in recipient rats without immunosuppressive treatment. Therefore, we first investigated how to regenerate rat nephrons in immunodeficient mice to avoid rejection (Fig. 2a). We used NOD/Shi-scid, IL-2RγKO Jic mice (NOG mice) as host immunodeficient mice^18^. We injected rat GFP-RPCs and DT below the renal capsule of the E13.5 MNs of the Six2-iDTR mice (Fig. 2b,c). The injected MNs were not separated from the ureters and bladder but were instead isolated with these components; these connected components were referred to as MNs with bladder (MNB; Fig. 2d). The Six2-iDTR mouse MNBs containing DT-ineffective exogenous rat RPCs were transplanted into the vicinity of the aorta of an adult NOG mouse (Fig. 2e). GFP expression was evaluated and identified 14 days after transplantation (Fig. 2f). The MNB was recovered and prepared as frozen sections (Fig. 2g, left), which were then subjected to haematoxylin–eosin (HE) staining (Fig. 2g, right) and immunostaining (Fig. 2h).

As a result, rat GFP-NPC-derived glomeruli (Fig. 2g, arrowhead) and tubules (Fig. 2g, arrow) formed within the transplanted Six2-iDTR MNs. GFP-positive regenerated glomeruli had nephrin-positive epithelial cells lining the CD31-positive vascular endothelial cells (Fig. 2h, lower). We also identified megalin-positive neo-proximal tubules and E-cadherin-positive neo-distal tubules (Fig. 2h, upper). As described above, we were able to regenerate interspecies chimeric kidneys in immunodeficient hosts by replacing the NPCs.

### Adjusting the immunosuppressive protocol for successful transplantation of regenerated interspecies chimeric kidneys

**Figure 3:**
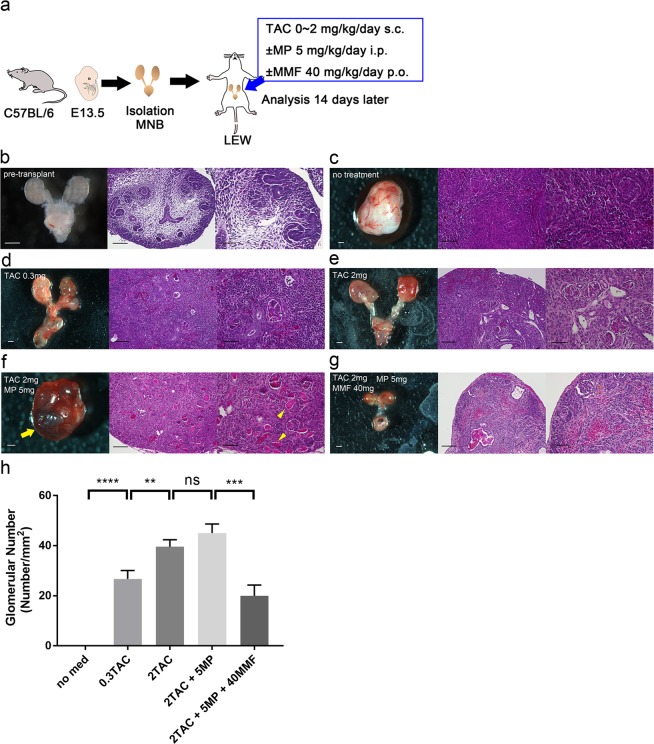
Optimisation of the immunosuppressive protocol for successful transplantation of mouse MNBs into rats. (**a**) Schematic of the experimental protocol. (**b**) Pre-transplanted MNB of the E13.5 mouse embryo. We observed immature nephrons at several developmental stages in the pre-transplanted MNs from the vesicles to the S-shaped bodies. (**c**) No TAC treatment. Fourteen days after transplantation, normal renal structures were not recognised due to severe inflammatory cell infiltration (*n* = 8 MNBs from two independent experiments). (**d**) Treatment with 0.3 mg/kg/day s.c. of TAC. Renal features remained, but glomeruli and tubule destruction and severe inflammatory cell infiltration were observed (*n* = 8 MNBs from two independent experiments). (**e**) Treatment with 2 mg/kg/day s.c. of TAC: the MNs showed normal renal features with minor inflammatory cell infiltration, but urine production was not confirmed (*n* = 8 MNBs from two independent experiments). (**f**) Treatment with 2 mg/kg/day s.c. of TAC + 5 mg/kg/day i.p. of MP: the MNs showed normal renal features, and what seemed to be urine production was confirmed in some specimens. The arrow indicates the embryonic bladder, which is filled with liquid. The arrowheads suggest urine production (*n* = 8 MNBs from two independent experiments). (**g**) Treatment with 2 mg/kg/day s.c. of TAC + 5 mg/kg/day i.p. of MP + 40 mg/kg/day p.o. of MMF. Few glomeruli remained, suggesting that MMF suppressed the development of MNs (*n* = 7 MNBs from two independent experiments). (**h**) The number of glomeruli per unit area was significantly higher in the 2 mg/kg/day of TAC group and the 2 mg/kg/day of TAC + 5 mg/kg/day of MP group than that in the other groups (*n* = 16, 16, 16, 16, 14 of MNs for each group, respectively). **P < 0.01; ***P < 0.001; ****P < 0.0001; ns, not significant. Error bars in bar plots represent the standard errors of the mean. (**b**–**g**) Left column: stereomicrographs of MNB (scale bars 500 µm), middle column: HE staining of MN at low magnification (scale bars 100 µm), right column: HE staining of MN at high magnification (scale bars 50 µm).

To successfully transplant regenerated chimeric kidneys into recipient rats, we examined the optimal immunosuppressive protocol for the successful transplantation of mouse MNBs into rats. We isolated MNBs from E13.5 B6 mouse embryos and transplanted them into adult Lewis rats. The host rats were treated with each immunosuppressive protocol. The MNBs were analysed 14 days later (Fig. 3a). We observed immature nephrons at several developmental stages in the pre-transplanted MNs from the vesicles to the S-shaped bodies (Fig. 3b). Fourteen days after transplantation with no immunosuppressive treatment, normal renal structures were unrecognisable because of severe inflammatory cell infiltration. The transplanted MNs appeared more like lymph nodes than kidneys (Fig. 3c). After tacrolimus (TAC: 0.3 mg/kg/day) treatment, the renal features remained, but destruction of the glomeruli and tubules and severe inflammatory cell infiltration were observed (Fig. 3d). With 2 mg/kg/day of TAC treatment, the MNs showed normal glomeruli and tubules with minor inflammatory cell infiltration, but urine production was not confirmed (Fig. 3e). To strengthen immunosuppression, we added methylprednisolone (MP: 5 mg/kg/day) to the 2 mg/kg/day of TAC. The MNs also showed normal glomeruli and tubules, and what appeared to be urine production was confirmed in some specimens, although it could not be validated because the samples were very small (Fig. 3f). After 2 mg/kg/day of TAC + 5 mg/kg/day of MP + mycophenolate mofetil (MMF: 40 mg/kg/day) treatment, few glomeruli remained, which suggests that MMF suppresses metanephric development (Fig. 3g). The number of glomeruli per unit area was significantly higher in the 2 mg/kg/day of TAC group and the 2 mg/kg/day of TAC + 5 mg/kg/day of MP group than that in the other groups (0 ± 0, 26.7 ± 3.4, 39.6 ± 2.8, 45.0 ± 3.7 and 20.0 ± 4.3 in no treatment, 0.3 mg/kg/day of TAC, 2 mg/kg/day of TAC, 2 mg/kg/day of TAC + 5 mg/kg/day of MP and 2 mg/kg/day of TAC + 5 mg/kg/day of MP + 40 mg/kg/day of MMF groups, respectively; Fig. 3h). We determined that the optimal immunosuppressive protocol was 2 mg/kg/day of TAC + 5 mg/kg/day of MP.

### *In vivo* regeneration of interspecies chimeric kidneys and analysis of neo-kidney function

**Figure 4:**
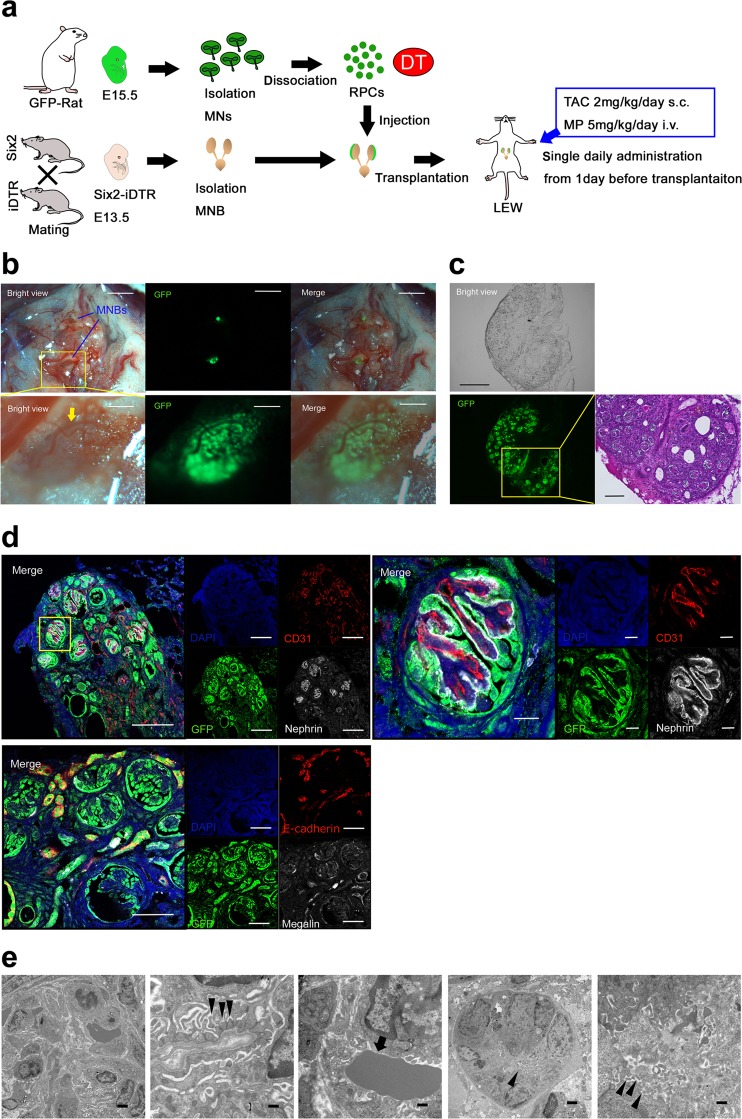
Regeneration of nephrons derived from transplanted rat NPCs in an adult Lewis rat with TAC and MP treatment. (**a**) Schematic of the experimental protocol. (**b**) E13.5 Six2-iDTR mouse MNB-containing rat RPCs and DT was transplanted into the vicinity of the aorta of an adult Lewis rat with TAC (2 mg/kg/day s.c.) and MP (5 mg/kg/day i.p.) treatment. Twenty-one days after transplantation, GFP expression was evaluated and identified (scale bars, upper column: 2 mm, lower column: 250 µm). The arrows represent the infiltration of host blood vessels. (**c**) Frozen sections were prepared from the collected MNB. We observed GFP-positive glomerular and tubular structures (left and middle column: scale bars, 300 µm). HE staining demonstrated that the glomeruli and tubules had regenerated from the transplanted rat NPCs (right column; scale bar, 100 µm). (**d**) Frozen sections were analysed by immunostaining. We identified neo-glomeruli (left upper column; scale bars,100 µm) and tubules (left lower column; sale bars, 50 µm) that originated from transplanted rat GFP-NPCs. Glomeruli had nephrin-positive epithelial cells lining CD31-positive vascular endothelial cells (right upper column; scale bar, 10 µm). (**e**) Aligned podocytes and a glomerular basement membrane in the neo-glomeruli were observed by transmission electron microscopy (left column, second from left). Slit diaphragm structures were observed (second from left arrowheads), and red blood cells could be confirmed in the neo-glomeruli (middle column, arrow). Neo-tubules displayed brush borders (second from right, right column arrows). Scale bars: left column, 2 µm; second from left, 500 nm; middle column, 500 nm; second from right, 2 μm; right column, 500 nm. Representative images of six specimens from three independent transplantation experiments are indicated.

**Figure 5:**
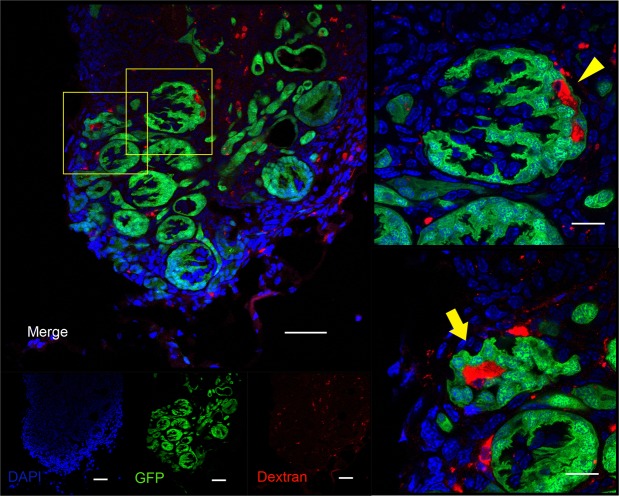
Analysis of neo-glomerular filtration. We injected low-molecular-weight, tetramethylrhodamine-labelled dextran into host rats. The accumulation of dextran in Bowman’s space of GFP-positive glomeruli (arrowhead) and in the lumen of GFP-positive tubules (arrow) was confirmed by immunostaining, demonstrating glomerular filtration (scale bars, left column: 50 µm, right upper and right lower column: 10 µm). Representative images of two specimens from one transplantation experiment are indicated.

Based on the above experiments, we investigated whether *in vivo* regeneration of interspecies chimeric kidneys is possible under optimal immunosuppression. The Six2-iDTR mouse MNB that contained rat GFP-RPCs and DT was transplanted into the vicinity of the aorta of an adult Lewis rat that was administered 2 mg/kg/day s.c. of TAC and 5 mg/kg/day i.p. of MP (Fig. 4a). GFP expression was evaluated and identified 21 days after transplantation. We observed host blood vessel infiltration into GFP-positive MNs (Fig. 4b). The MNB was recovered and prepared as frozen sections (Fig. 4c, left), which were then subjected to HE staining (Fig. 4c, right) and immunostaining (Fig. 4d). In the same manner as when transplanted into NOG mice, we identified regenerated glomeruli and tubules originating from transplanted rat GFP-NPCs (Fig. 4c,d). GFP-positive regenerated glomeruli comprised nephrin-positive epithelial cells lining CD31-positive vascular endothelial cells (Fig. 4d, upper). We used the anti-vimentin monoclonal antibody V9 for species-specific detection in rats but not mice^19^; CD31-positive vascular endothelial cells were stained with rat-specific anti-vimentin antibodies. In other words, neo-glomeruli were vascularised by blood vessels originating from the host Lewis rat, not Six2-iDTR mouse (Supplementary Fig. S2). Electron microscope observation of the regenerated glomeruli showed slit diaphragm structures that were composed of aligned podocytes and basement membranes (Fig. 4e, left and second from left). The presence of erythrocytes within the neo-glomeruli was observed (Fig. 4e, middle). Renal tubule brush borders were also detected (Fig. 4e, right and second from right). To determine whether regenerated glomerular filtration was present, we injected tetramethylrhodamine-labelled 10-kDa dextran into the recipient rat through the tail vein one hour prior to collecting the regenerated MNs. Histology revealed the presence of dextran in Bowman’s space of GFP-positive glomeruli and in the lumen of GFP-positive tubules (Fig. 5), suggesting a functional connection between neo-glomeruli and host vessels; this implies the presence of neo-glomeruli filtration and urinary flow to the neo-tubules. Using the NPC replacement system, we successfully regenerated interspecies kidneys that developed a vascular system *in vivo* under optimal immunosuppression conditions.

## Discussion

Our ultimate goal is to regenerate human stem cell-derived kidneys that are functional *in vivo* to establish a new alternative therapy to dialysis and kidney transplantation. In this study, we examined the possibility of the *in vivo* regeneration of interspecies kidneys using NPC replacement; our aim is to apply this method to humans in the future. We attempted to generate rat/mouse interspecies chimeric kidneys that can function *in vivo*.

The principle of our method involves regenerating nephrons by replacing Six2-positive host cells with exogenous donor cells. Six2 is a transcriptional factor that is expressed in the existing cap mesenchymal cells around the UB. Six2-positive cells are considered NPCs and differentiate into nephrons consisting of podocytes, proximal tubules, the loop of Henle, and distal tubules^20^. Conversely, the collecting duct system and interstitium have a different origin. Importantly, we previously revealed that nephrons derived from rat NPCs could connect to the host mouse collecting ducts, even though the nephrons and collecting ducts were heterogeneous^17^. This result is the foundation for the prediction that regenerated kidneys through an NPC replacement system are capable of functioning *in vivo* even when MNs belong to a different species.

To establish *in vivo* regeneration, we injected NPCs-containing rat RPCs and DT into the nephrogenic zone of the Six2-iDTR MNs and transplanted them into the vicinity of the aorta in an adult rat. In this case, the collecting duct system and interstitium are xenogeneic to the recipient and will be rejected, even though the regenerated nephrons and vascular system are allogenic. Therefore, optimal immunosuppression that affects the generation of nephrons as little as possible and can sufficiently suppress rejection is necessary. This is demonstrated in the following examination. Firstly, we generated rat nephrons in immunodeficient mice to avoid the rejection of regenerated chimeric kidneys. Secondly, we examined the optimal immunosuppressive protocol for the successful transplantation of regenerated chimeric kidneys into recipient rats. Finally, we aimed to regenerate rat nephrons in recipient rats that were treated with optimal immunosuppressants. As a result, we developed regenerated rat nephrons using the Six2-iDTR mouse MNs as a scaffold in the recipient rats that received the optimal immunosuppressive therapy. We confirmed a functional connection between the neo-glomeruli and host vessels and functional neo-glomeruli filtration. This study suggested that xenogeneic embryonic kidneys were a useful bioreactor for kidney regeneration using an NPC replacement system.

Conveniently, MNs, which are foetal organs, may have a low immunogenicity and thus are suitable for transplantation. First, a developing MN is expected to be depleted of dendritic antigen-presenting cells because there would be no mature dendric cells at this stage of embryonic development^21^. Second, there would be a reduced expression of donor antigens such as HLA class I and II on a developing MN compared to an adult graft^22^. Third, a transplanted MN would be supplied by blood vessels originating from the host^23^. In fact, the immune advantage of a developing MN over a developed adult kidney has been demonstrated in a direct comparison of xenotransplantation into host animals treated with immunosuppressants^24^. We believe that our method, in which MNs are used as a regeneration scaffold, is also advantageous in terms of immune response. Successful cases of MN xenotransplantation by co-stimulatory blockade have been reported in previous studies^25,26^. In the present study, we aimed to engraft regenerated kidneys with immunosuppressants used in general clinical practice. In a previous study, rat/mouse interspecies adult kidney xenografts with calcineurin inhibitor monotherapy were completely rejected within a few days^27^, whereas in our observation, metanephric xenografts with TAC monotherapy could avoid rejection to some extent. A previous study investigating the effects of various immunosuppressants in allogeneic metanephric transplantation has shown that TAC and MP had no effect on the development of MNs, whereas MMF inhibited their development^28^. In our examination, MN growth was also poor after MMF administration. In conclusion, regenerated kidney engraftment and growth occurred after TAC and MP administration.

The advantage of this method is the ability to maintain the three-dimensional kidney structure, including the urinary drainage system. In this study, urine production was not confirmed in the mouse-rat model because of the very small samples, although neo-glomeruli filtration was validated. If this system is applied to a larger animal and control of rejection is enough, urine production may be confirmed, and the construction of a urinary excretion pathway may be possible using the method we previously reported^29^.

To regenerate human kidneys, this system will be applied to pigs, whose organ sizes are similar to those of humans. In brief, NPCs derived from human iPSCs may be injected into the MNs of porcine foetuses that are genetically manipulated to have an NPC elimination system. Human nephrons may regenerate in porcine MNs by eliminating the porcine NPCs and replacing them with human NPCs, thus creating a chimeric human/pig kidney. The regenerated kidney will be transplanted into patients with end-stage renal disease, and an excretion pathway will be constructed. Recently, other researchers reported that they achieved human/pig chimera formation by transplanting human iPSCs into porcine blastocysts^30^, suggesting the possibility of generating human/pig chimera kidneys. It is not difficult to supply a cell source because protocols for inducing NPCs from human iPSCs have been developed^5–7^; additionally, the expansion culture of NPCs is possible^31,32^.

Recently, there has been remarkable progress in xenotransplantation because of the genetic engineering advances in pigs and advancement in immunosuppression strategies. The use of multi-transgenic pigs, including α1,3-galactosyltransferase gene knockout (GalT-KO) pigs, has facilitated the survival of life-supporting kidneys for >6 months in pigs and non-human primate models^33,34^. However, nephrotic syndrome remains a common complication following kidney xenotransplantation and is associated with vascular thrombosis and infections that may limit the survival of recipients^35^. Whether nephrotic syndrome results from immune injuries or physiological incompatibilities between species, or both, remains unclear; recent experiments attribute its occurrence to a continuing immune response^36^. Whichever the cause, replacing pig nephrons with human cells may solve this problem. Reportedly, substantial suppression required to avoid the rejection of xenografts could cause serious infections in recipients^33^. Furthermore, replacing nephrons, which are one of the targets to rejection, could decrease immunosuppression, although it warrants further investigation in the next step.

Before utilizing the NPC replacement system in humans, some significant challenges need to be addressed. First being immunogenicity due to the contamination of host pig-derived cells that cannot be entirely eliminated. Second is the risk of cross-species transmission of porcine endogenous retroviruses (PERVs). Reportedly, various multi-transgenic pigs lacking immunogenicity have been developed till date^33,34^. A study recently reported the successful production of PERV-inactivated pigs using CRISPER-Cas9^37^. To overcome these challenges, we may also need to use such genetically modified pigs as bioreactors using our methods.

As a fundamental problem, the iDTR system cannot be applied directly to humans because human cells permanently express DTR. Therefore, human cells undergo apoptosis when treated with DT. We have begun to investigate a new model to ablate Six2-positive NPCs using an alternative drug that does not affect human cells.

In conclusion, we regenerated rat nephrons using Six2-iDTR MNs as a scaffold in recipient rats treated with the optimal immunosuppressive drugs. Therefore, we demonstrated the *in vivo* regeneration of interspecies chimeric kidneys using an NPC replacement system. This method will be useful for research on human kidney regeneration and to establish an *in vivo* disease model. Furthermore, this method will be also useful for *in vivo* drug screening and toxicity analyses. More importantly, this novel strategy may represent an effective method for kidney regeneration. In the future, we will aim to regenerate human nephrons in pig MNs that are genetically modified to possess a progenitor cell elimination system using NPCs derived from human-induced iPSCs and the platform described in this study.

## Methods

### Mouse and rat maintenance and experiments

Pregnant Sprague-Dawley-Tg (CAG-EGFP) rats (GFP-rats, 10–15 weeks old, E15.5), C57BL/6JJmsSlc mice (B6 mice, 10–15 weeks old, E13.5), and adult male LEW/SsNSlc rats (Lewis rats, 10–11 weeks old) were purchased from SLC Japan (Shizuoka, Japan). Adult male NOD/Shi-scid, IL-2RγKO Jic mice (NOG mice, 8–10 weeks old) were purchased from CLEA Japan (Tokyo, Japan). C57BL/6-Gt (ROSA) 26Sor [tm1(HBEGF)Awai]/J mice (iDTR mice) were purchased from Jackson Laboratory (Bar Harbor, ME, USA). The iDTR system was designed to express Cre-inducible DTR on specific cell populations and ablate them by administering DT^38^. Six2-GFP-Cre transgenic mice (Six2 mice) were a gift from McMahon^20^. The Six2 mice were crossed with iDTR mice to obtain offspring (Six2-iDTR mice). The mice were bred using timed mating; 12 PM on the day of vaginal plug detection was considered 0.5 days post coitum. Both male and female mice were used at 10–25 weeks of age. All mice and rats were housed in a temperature- and humidity-controlled environment with a 12-hour light-dark cycle; they were provided with standard laboratory chow and water.

### NPCs-containing RPCs derived from the enzymatic dissociation of MNs

MNs (E15.5) were prepared from GFP-rats. MN dissociation was performed as previously described^39^. Briefly, pregnant rats were anaesthetised with isoflurane (Pfizer, New York, NY, USA) inhalation. The embryos were harvested, and the pregnant rats were then killed immediately by an infusion of pentobarbital (120 mg/kg: Kyoritsu Pharma, Tokyo, Japan). All the embryos were killed by decapitation. The MNs were dissected in α-minimum essential medium (α-MEM; Life Technology, Grand Island, NY, USA) under a surgical microscope. The MNs were then collected in 1 mL of prewarmed (37 °C) Accutase (Innovative Cell Technologies, San Diego, CA, USA) and incubated at 37 °C for 15 min, with manual pipetting every 5 min to aid digestion. Next, the MNs were centrifuged at 300 × *g* for 5 min. The pellets were resuspended in 1 mL of phosphate-buffered saline (PBS; Gibco Life Technologies) and manually dissociated to single-cell suspensions. The cells were then passed through a 40-μm cell strainer (Corning Inc., Corning, NY, USA) to remove any remaining clumps and were centrifuged at 700 × *g* for 3 min. The pellets were resuspended in 200 µL of DT (Wako, Osaka, Japan) solution (DT was dissolved in PBS at 0.1 ng/µL) and centrifuged at 700 × *g* for 3 min. The supernatant was completely removed. The tubes were tapped to mix the pellets and were incubated on ice until use.

### Injection of RPCs into the nephrogenic zone

RPC injection was performed as previously described elsewhere^17^. Briefly, Six2-iDTR mice that were 13.5 days pregnant were killed by dislocation of the cervical vertebrae. The foetuses were harvested and then placed in 10-cm dishes containing Hanks balanced salt solution (HBSS; Life Technologies). The foetuses were killed by decapitation. After the pair of MNs became visible in the body, the foetuses were fixed with microtweezers. Subsequently, a glass needle filled with RPCs and DT, which were prepared as described above, was inserted into the renal membrane from the renal hilus, and the cells were injected under the renal capsule by mouth pipetting (Drummond Scientific Company, Broomall, PA, USA) under the fluorescence stereo microscope (Leica M205FA, Leica Microsystems, Wetzlar, Germany). The injected MNBs were detached from the foetuses and then subjected to organ culture or were transplanted into NOG mice and LEW rats.

### Organ culture of isolated MNs

Organ cultures of isolated MNs were performed as previously reported^40^. Briefly, isolated MNs of E13.5 Six-iDTR mouse embryos were placed at the air−fluid interface on a polycarbonate filter with an average pore size of 0.4 µm (12-mm Transwell; Corning). The medium was α-MEM supplemented with 20% foetal bovine serum (FBS; Hyclone, Logan, UT, USA) and 1% Antibiotic-Antimycotic (Life Technologies). The organs were cultured for five days at 37 °C with 5% CO_2_. One hundred microliters of DT solution (10 ng/well) was then added to the MN culture medium daily for five days after changing the medium.

### Transplantation of MNB

MNB transplantation was performed as previously reported^29^. NOG mice or Lewis rats were used as the host animal. The host mice were anaesthetised with normal saline containing 0.3 mg/kg medetomidine (Zenoaq, Fukushima, Japan), 4.0 mg/kg midazolam (Astellas Pharma, Tokyo, Japan), and 5.0 mg/kg butorphanol (Meiji Seika, Tokyo, Japan). The host rats were anaesthetised by isoflurane inhalation. Isolated MNBs from E13.5 Six2-iDTR or B6 mouse embryos were transplanted under the retroperitoneal vicinity of the descending aorta using a surgical microscope (Leica S9D, Leica Microsystems). After two to three weeks, the recipients were killed by intraperitoneal pentobarbital infusion, and the transplanted MNBs were removed for histological analysis.

### *In vivo* bioluminescence imaging

Some recipient rats were intravenously administered 200 µL of 5 mg/mL tetramethylrhodamine-labelled dextran (10,000 MW) (D1817; Thermo Fisher Scientific, Waltham, MA, USA) one hour prior to collecting the regenerated MNs^31,41^. The collected specimens were analysed by immunostaining.

### Immunosuppression

The following immunosuppressive agents were used to treat the recipient rats one day before transplantation. Tacrolimus (0.3–2 mg/kg/day; Astellas Pharma) was administered subcutaneously. Methyl prednisolone (5 mg/kg/day; Pfizer) was administered by intraperitoneal injection. Mycophenolate mofetil (40 mg/kg/day; Chugai Pharma, Tokyo, Japan) was administered orally by gavage.

### HE staining

HE staining was performed as described elsewhere^29^. Tissues grown from implanted MNBs were fixed with 4% paraformaldehyde in PBS, dehydrated in graded alcohols, embedded in paraffin, cut into 4-µm sections, and stained with HE. Each section was assessed by microscopy (BZ-9000; Keyence, Osaka, Japan).

### Glomerular number

We counted glomeruli in the hematoxylin–eosin-stained MNs post-transplantation and determined the sectioned area. Then, the glomerular number was divided by the area of the section to attain the glomerular number per unit area (pieces/mm^2^). Two researchers counted the number of glomeruli in this study.

### Immunostaining

Immunostaining was performed as previously described^39,40^. The samples were fixed with 4% paraformaldehyde in PBS for 60 min, dehydrated with 20% sucrose in PBS, embedded in optimal cutting temperature (OCT) compound (Sakura Finetechnical, Tokyo, Japan), and cryosectioned at 8-µm thickness. After blocking for one hour at room temperature, the sections were incubated overnight at 4 °C with primary antibodies (a list of primary antibodies is summarised in Table 1); consequently, they were incubated at room temperature with secondary antibodies conjugated with Alexa Fluor 488, 546 and 647 for one hour. The sections were mounted with DAPI-containing Prolong-Gold mounting medium (Thermo Fisher Scientific). To visualise the tetramethylrhodamine-labelled dextran by fluorescence microscopy, OCT embedded sections were washed with PBS and directly mounted with DAPI-containing Prolong-Gold mounting medium^41^. Each section was examined under a fluorescence microscope (LSM880 confocal; Carl Zeiss, Munich, Germany).

**Table 1:** List of primary antibodies.

Antigen	Host	Supplier	Cat. No.	Dilution
Six2	Rabbit	Proteintech	1156-AP	1:100
Cytokeratin-8	Rat	DSHB	TROMA-IC	1:100
E-cadherin	Mouse	BD Biosciences	BD610181	1:100
CD31	Rabbit	Abcam	ab2864	1:50
Megalin	Goat	Santa Cruz Biotechnology	sc-1478	1:100
Nephrin	Goat	Santa Cruz Biotechnology	sc-19000	1:100
GFP	mouse	MBL	MBL048-3	1: 100
GFP	rabbit	MBL	MBL598	1:100
Vimentin	Mouse	Abcam	ab8069	1:100

### Electron microscopy

For the electron microscopy observation, the specimens were fixed by immersion in 2% glutaraldehyde in 0.1 M phosphate buffer overnight at 4 °C and post-fixed in 1% osmium tetroxide in the same buffer at 4 °C for two hours. After dehydration in ethanol, the samples were placed in propylene oxide and subsequently embedded in Epok 812 (Oken, Tokyo, Japan). Ultrathin sections were cut with a diamond knife, stained with uranyl acetate and lead citrate and observed under a H-7500 electron microscope (Hitachi, Tokyo, Japan) at an accelerating voltage of 80 kV.

### Statistical analyses

In this study, means were compared using the unpaired two-tailed Student’s *t*-test. All statistical analyses were performed using the PRISM7 software (GraphPad Software, La Jolla, CA, USA). We considered *P* values of < 0.05 as statistically significant.

### Ethical considerations

All animal experiments were approved by the Institutional Animal Care and Use Committee and the Recombinant gene Research Safety Committee of the Jikei University School of Medicine (Permit Number: 2017-041C1, 29–36). These experiments were conducted in conformity with the NIH Guide for the Care and Use of Laboratory Animals. All efforts were made to minimise animal suffering.

## Supplementary information

Supplementary Information

## Data Availability

The datasets generated during the current study are available from the corresponding author on reasonable request.
