# Supplementary material for: In vivo regeneration of interspecies chimeric kidneys using a nephron progenitor cell replacement system
Source: Sci Rep. 2019 May 6;9:6965. doi: 10.1038/s41598-019-43482-2 (PMC6502858; doi:10.1038/s41598-019-43482-2)
Supplement: Supplementary file 1 — Supplementary Information [file 41598_2019_43482_MOESM1_ESM.pdf]

Supplementary Information

In vivo regeneration of interspecies chimeric kidneys using a nephron progenitor cell replacement system

Authors

Toshinari Fujimoto[1,2], Shuichiro Yamanaka[1], Susumu Tajiri[1], Tsuyoshi Takamura[1,2], Yatsumu Saito[1], Kei Matsumoto[1], Kentaro Takase[1,3], Fukunaga Shohei[3], Hirotaka James Okano[2], Takashi Yokoo[1*]

[1]Division of Nephrology and Hypertension, Department of Internal Medicine, The Jikei University School of Medicine, 3-25-8, Nishi-Shimbashi, Minato-ku, Tokyo, 105-8461, Japan

[2]Division of Regenerative Medicine, The Jikei University School of Medicine, 3-25-8, Nishi-Shimbashi, Minato-ku, Tokyo, 105-8461, Japan

[3]Division of Cardiology and Nephrology, Department of Internal Medicine, Shimane University Faculty of Medicine, Izumo, Shimane 693-8501, Japan

***Correspondence**

Prof. Takashi Yokoo, MD, PhD

Division of Nephrology and Hypertension, Department of Internal Medicine, The Jikei University School of Medicine, 3-25-8, Nishi-Shimbashi, Minato-ku, Tokyo, 105-8461, Japan

Tel: +81-3-3433-1111; Fax: +81-3-3433-4297; E-mail: tyokoo@jikei.ac.jp

a

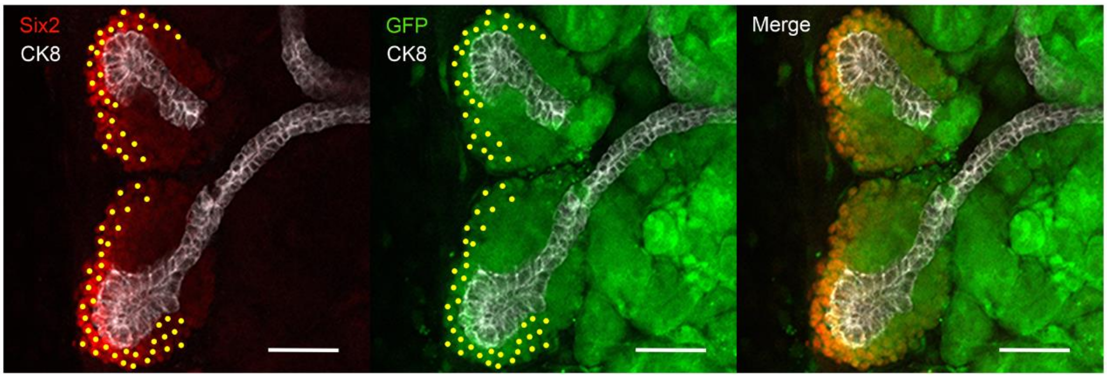

b

Measurement of the replacement rate in the cap mesenchyme		
Six2-positive NPCs	GFP-NPCs (Donor cells)	Percentage of GFP cells in the cap mesenchyme
29.8 ± 2.5	29.8 ± 2.5	100 ± 0 (%)

Supplementary Figure S1. The ratio of donor nephron progenitor cells (NPCs) in the Six2-positive domain after NPC replacement *in vitro*

(a) Representative images of the cap mesenchyme after eliminating host NPCs and replacing with donor rat GFP-NPCs. We manually counted the ratio of donor NPCs in the Six2-positive domain. Left column, yellow spots indicate Six2-positive NPCs; middle column, these spots entirely merged with donor GFP-NPCs; right column, the merged image (scale bars, 50 μm). **(b)** We assessed the number of donor GFP-NPCs and the replacement rate in the cap mesenchyme. The number of GFP-NPCs was 29.8 ± 2.5 (mean ± standard errors of the mean; n = 6); 100% replacement of host NPCs with donor NPCs was possible.

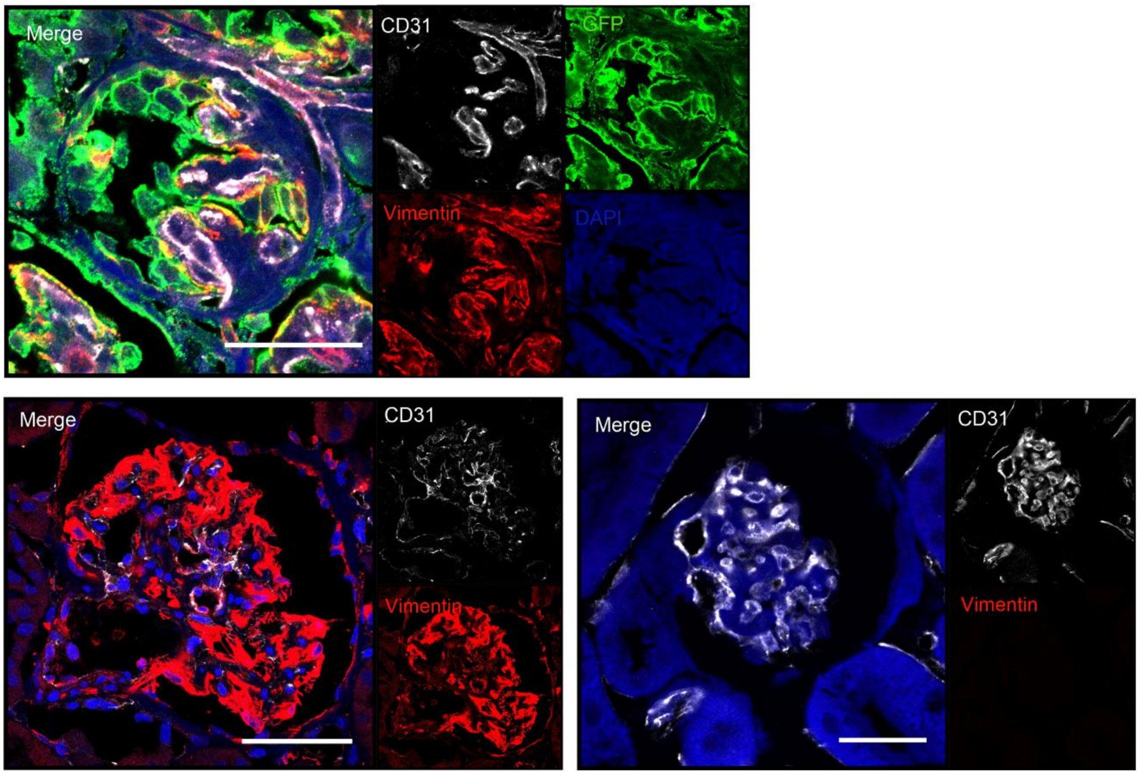

Supplementary Figure S2. Neo-glomeruli were vascularised with blood vessels originating from the host rat

We stained the sections of the regenerated kidney, native rat kidney and native mouse kidney with anti-CD31 and anti-vimentin antibodies. Endothelial cells in neo-glomeruli were expressed as the endothelial marker CD31 and the rat-specific marker vimentin V9 (upper column; scale bars, 100 μm). In addition, endothelial cells in rat glomeruli were expressed as the endothelial marker CD31 and the rat-specific marker vimentin V9 (left lower column; scale bars, 50 μm). Endothelial cells in mouse glomeruli were expressed as the endothelial marker CD31 but not as the rat-specific marker vimentin V9 (right lower column; scale bars, 50 μm).
